# Habitat-specific patterns of bacterial communities in a glacier-fed lake on the Tibetan Plateau

**DOI:** 10.1093/femsec/fiae018

**Published:** 2024-02-20

**Authors:** Xuezi Guo, Qi Yan, Feng Wang, Wenqiang Wang, Zhihao Zhang, Yongqin Liu, Keshao Liu

**Affiliations:** State Key Laboratory of Tibetan Plateau Earth System, Environment and Resources (TPESER), Institute of Tibetan Plateau Research, Chinese Academy of Sciences, Beijing 100101, China; University of Chinese Academy of Sciences, Beijing 100049, China; Center for the Pan-Third Pole Environment, Lanzhou University, Lanzhou 730000, China; State Key Laboratory of Tibetan Plateau Earth System, Environment and Resources (TPESER), Institute of Tibetan Plateau Research, Chinese Academy of Sciences, Beijing 100101, China; Center for the Pan-Third Pole Environment, Lanzhou University, Lanzhou 730000, China; State Key Laboratory of Tibetan Plateau Earth System, Environment and Resources (TPESER), Institute of Tibetan Plateau Research, Chinese Academy of Sciences, Beijing 100101, China; University of Chinese Academy of Sciences, Beijing 100049, China; State Key Laboratory of Tibetan Plateau Earth System, Environment and Resources (TPESER), Institute of Tibetan Plateau Research, Chinese Academy of Sciences, Beijing 100101, China; Center for the Pan-Third Pole Environment, Lanzhou University, Lanzhou 730000, China; State Key Laboratory of Tibetan Plateau Earth System, Environment and Resources (TPESER), Institute of Tibetan Plateau Research, Chinese Academy of Sciences, Beijing 100101, China

**Keywords:** bacterial communities, co-occurrence network, glacier-fed lake, habitat heterogeneity, microbial source tracking

## Abstract

Different types of inlet water are expected to affect microbial communities of lake ecosystems due to changing environmental conditions and the dispersal of species. However, knowledge of the effects of changes in environmental conditions and export of microbial assemblages on lake ecosystems is limited, especially for glacier-fed lakes. Here, we collected water samples from the surface water of a glacier-fed lake and its two fed streams on the Tibetan Plateau to investigate the importance of glacial and non-glacial streams as sources of diversity for lake bacterial communities. Results showed that the glacial stream was an important source of microorganisms in the studied lake, contributing 45.53% to the total bacterial community in the lake water, while only 19.14% of bacterial community in the lake water was seeded by the non-glacial stream. Bacterial communities were significantly different between the glacier-fed lake and its two fed streams. pH, conductivity, total dissolved solids, water temperature and total nitrogen had a significant effect on bacterial spatial turnover, and together explained 36.2% of the variation of bacterial distribution among habitats. Moreover, bacterial co-occurrence associations tended to be stronger in the lake water than in stream habitats. Collectively, this study may provide an important reference for assessing the contributions of different inlet water sources to glacier-fed lakes.

## Teaser

This study demonstrated that among two major inlets a glacier-fed stream was more important than a non-glacier-fed stream for seeding the bacterial community of the lake.

## Introduction

Glacier-fed lakes, as an important part of the cryosphere, are aquatic ecosystems directly affected by glaciers and are sensitive indicators of climate change (Hotaling et al. 2017, Zhang et al. 2020). Global warming has been shown to increase both the rate and extent of glacier melting in high-latitude and high-altitude regions (Lee et al. 2017, Cauvy-Fraunie and Dangles 2019). Rapid melting of glaciers is expected to result in an expansion of glacier-fed lake areas due to increased meltwater (Slemmons et al. 2013, Zhang et al. 2015). Massive glacial meltwater inflows could affect microbial assemblages by exerting significant controls on abiotic and biotic features of glacier-fed lakes (Slemmons and Saros 2012, Tiberti et al. 2020). For instance, glacier meltwater can transport high concentrations of mineral particles into downstream ecosystems, leading to high turbidity in glacier-fed ecosystems (Sommaruga 2015). The high turbidity limits light penetration into water, causing unfavorable conditions for primary producers and further indirectly affecting the microbial community assemblages (Peter and Sommaruga 2016, Peter et al. 2018, Tiberti et al. 2020). Furthermore, glacial meltwater may also directly affect the microbial diversity of downstream lakes by exporting microorganisms (Cameron et al. 2017, Kohler et al. 2020).

In addition to the recruitment of microbes from glaciers, glacier-fed lakes also can receive species from surrounding environments, such as soil (Crump et al. 2012), permafrost meltwater (Bomberg et al. 2019) and groundwater (Echeverria-Vega et al. 2018). Given that glaciers are retreating rapidly and many will disappear within decades under global warming, the effects of glacier meltwater on microbial diversity in downstream lakes will diminish (Peter and Sommaruga 2016, Liu et al. 2019). Subsequently, the water and microbial sources of glacial lakes will shift to be dominated by non-glacial streams (e.g. groundwater-fed streams) (Freimann et al. 2013). Such shifts in water and microbial sources will have major impacts on the physicochemical characteristics and microbial communities in glacial lakes (Peter and Sommaruga 2016). In general, glacial lake systems are influenced by high discharge of glacial meltwater during summer ablation and an increasing influence of groundwater towards winter (Freimann et al. 2013). Liu et al. (2019) demonstrated that bacterial abundances and alpha diversity increased as the amount of meltwater increased during the glacier melting seasons. In contrast to glacial meltwater supply, groundwater-fed systems provide more spatiotemporal stability and a more homogeneous landscape (Brown et al. 2003, Battin et al. 2004), and the smaller fluctuations in physicochemical characteristics in such habitats cause reduced variability in bacterial communities. Taken together, it is important to evaluate the impact of glacial and non-glacial stream sources on microbial diversity in glacial lakes.

SourceTracker based on a Bayesian mixing model has been widely used to identify the sources of community assembly (Peter and Sommaruga 2016, Comte et al. 2017, Cameron et al. 2020). It is commonly recognized that microbial communities are highly unbalanced: a small number of species are highly abundant (referred to as “abundant biosphere”), while a large number of other species have a low abundance (referred to as the “rare biosphere”) (Lynch and Neufeld 2015, Li et al. 2023). The abundant microorganisms contribute most of the microbial biomass, whereas the rare microorganisms may act as a “seed bank” for maintaining microbial diversity. Previous studies have found that abundant and rare communities showed the opposite source patterns in “sink microbial communities”. For instance, a previous study on sediment bacteria in the Yarlung Tsangpo River showed that the potential contributions of abundant and rare communities from upstream and tributaries to downstream were different (Liu et al. 2022). By contrast, several studies have also shown that the total, abundant and rare communities have similar proportions of contributions for sink microbial communities (Wang et al. 2021, Xiong et al. 2021). However, it remains unclear whether the source patterns of abundant and rare taxa in the microbial communities of the glacier-fed lake are similar.

To evaluate the role of glacial and non-glacial sources for diversity of the glacier-fed lake, we simultaneously collected water samples from a glacier-fed lake named Amuco and its two external inlet streams on the Tibetan Plateau in June 2018, that is, a glacial stream and a non-glacial stream (Fig. S1). Bacterial communities were obtained using the Illumina MiSeq sequencing method with 16S rRNA gene amplicons. Considering the lake is supplied mainly by glacial meltwater, we hypothesized that the species pool of the glacial stream contributes more to the microbial communities of the lake than the non-glacial stream. Given that the glacier-fed lake acts as recipients of microorganisms from diversely allochthonous resources, we further hypothesized that the bacterial diversity in the glacier-fed lake was higher than that in the two external inlet streams.

## Materials and methods

### Study area and sampling

Lake Amuco (33.45°N, 88.72°E, 4960 m above sea level) is situated in the eastern Qiangtang Plateau of the central Tibetan Plateau. The area of the lake is approximately 34.8 km^2^ and the maximum depth is 19 m. Lake Amuco receives meltwater from two streams. One is a glacial stream formed by glacial meltwater from the Qiangtang No.1 glacier (33.29°N, 88.70°E) in the southern part of the lake with a total length of 12 km (Li et al. 2017). The other stream is a non-glacial stream in the northwestern part of the lake, which is formed by precipitation and permafrost meltwater (Fig. S1).

In June 2018, a total of 37 water samples were collected from the surface of the lake and its two inlet streams. Specifically, the 37 samples were collected from the surface (0.5 m) of the glacier-fed lake (13), the glacial stream (15) and the non-glacial stream (9), respectively. Approximately 5 L of water was collected from each site with a Schindler sampler. Each water sample was divided into two subsamples: one for DNA extraction and the other for measuring physiochemical properties. First, 500 ml of water was filtered through a 20-µm mesh (Millipore, USA) to remove large particles and was then filtered through a 0.22-µm polycarbonate membrane (47-mm diameter, Millipore, USA) for DNA extraction. The filters were stored at −80°C until molecular analysis. Also, 100 ml of the water sample was filtered through a 0.45-µm hydrophilic polyethersulfone (PES) syringe filter (25 mm, Anpel) to measure the dissolved organic carbon (DOC) and total nitrogen (TN) concentrations and was frozen at −20°C for physicochemical analysis in the laboratory.

Sampling site coordinates were recorded using a Global Positioning System. The pH, conductivity (Cond), total dissolved solids (TDS) and water temperature (Temp) were monitored in situ with a YSI multi-probe Water Quality Sonde (YSI EXO2, Yellow Springs, OH, USA). The DOC and TN concentrations in the filtered water were measured by a Shimadzu Total Organic Carbon Analyzer (TOC-VCPH, Shimadzu Corporation, Japan) with a TN measuring unit (TNM-1, Shimadzu, Japan) through high-temperature catalytic oxidation (680°C). Before combustion, each sample was first acidified with 1 M HCl and sparged with carrier gas to remove all the inorganic carbon (Guo et al. 2022).

### DNA extraction, 16S rRNA gene amplicon sequencing and Illumina MiSeq sequencing

Environmental DNA was extracted from the filters using the Fast DNA^®^ Spin kit (MP Biomedicals, Santa Ana, CA, USA) according to the manufacturer's instructions. The raw DNA was quantified with a NanoDrop 1000 Spectrophotometer (Thermo-Scientific). The V4 region of the bacterial 16S rRNA genes was amplified with a uniquely tagged primer pair 515F (5′GTGCCAGCMGCCGCGGTAA-3′) and 806R (5′GGACTACHVGGGTWTCTAAT -3′). The 50-µL polymerase chain reaction (PCR) systems were performed in triplicate, with each containing 10 ng of DNA template, 5 µL of 2x Premix Taq DNA polymerase (Takara Biotechnology, Dalian, China), 1 µL of each primer (10 µM) and 20 µL of nuclease-free water. PCR was performed under the following conditions: 94°C for 3 min, followed by 30 cycles of 94°C for 30 s, 60°C for 30 s and 72°C for 1 min, after which we performed a final cycle of 5 min at 72°C. After amplication, we pooled multiple samples together in equal volumes. Pooled samples were purified using Agencourt AMpure XP beads. PCR products were sequenced using the Illumina MiSeq platform 2 × 250 bp paired-ends (Illumina, San Diego, CA, USA). The raw sequencing data generated in this study were submitted to the NCBI short reads archive (SRA) database under BioProject number PRJNA884020.

### Sequence processing

The paired-end reads were assembled with FLASH (v. 1.2.11) using default settings (Magoc and Salzberg 2011). We processed the sequences mainly using the Quantitative Insights Into Microbial Ecology (QIIME) pipeline (v. 1.8) (Caporaso et al. 2010). After quality filtering, denoising and chimera removal, high-quality reads were clustered into operational taxonomic units (OTUs) at the cutoff of 97% with the UPARSE algorithm (Edgar 2013). Representative sequences from each OTU were determined using the reference SILVA database (version 132 NR) at a confidence cutoff of 80% (Quast et al. 2013). After taxonomies had been assigned, OTUs that were affiliated with chloroplast, archaeal and unclassified sequences were removed from the following analysis. To avoid artifacts from sequencing depth, we used a randomly selected subset of 20 600 sequences based on the sample with the smallest sequencing for further analysis.

### Statistical analysis

The Alpha-diversity indices (Shannon diversity, Richness, Pielou's evenness) were calculated with the package “vegan” in R (version 4.0.3). Non-metric multidimensional scaling analysis (NMDS) based on the Bray–Curtis dissimilarity was applied to characterize differences in bacterial community composition between samples (Clarke 1993). Analysis of similarities (ANOSIM) analyses of Bray–Curtis dissimilarity was used to evaluate the differences between samples grouped by habitats. Similarity percentage (SIMPER) analysis identified the principal OTUs responsible for the differences between sample groups, which can be performed in the PAST software (Hammer et al. 2001). The significantly discriminant taxa in each major cluster (habitats groups) were determined using the Linear discriminant analysis (LDA) effect size (LEfSe) method (Segata et al. 2011). The Venn diagram was drawn using the “VennDiagram” package (Chen and Boutros 2011).

To identify the significance of environmental factors that may be influencing changes in community composition, a distance-based multivariate linear model (DistLM) analysis on Bray–Curtis distances was performed using the DISTLM_forward3 program (McArdle and Anderson 2001). And we assessed the impact of environmental factors on the indicator species by Randomforest analysis, which was performed with the package “randomForest” in R (version 4.0.3).

SourceTracker based on a Bayesian mixing model (Knights et al. 2011) was used to identify the different sources and estimate their contribution to the bacterial community composition of the lakes. It could estimate the relative contributions of microbes from multiple sources to an environment. In SourceTracker analyses, the relative contributions from different sources to a sink environment are modeled as a probabilistic mixture of the composition of sources (Baral et al. 2018). Independent SourceTracker analyses were carried out for different taxonomic community levels (total community, abundant community and rare community) and dominant bacterial phyla/classes a total of 14 times. We defined “abundant OTUs” as those having relative abundances above 0.1% of total sequences, and “rare OTUs” as those having relative abundances below 0.01% (Jiao et al. 2017, Jiao and Lu 2020).The surface water of the lake was set as the sink, and the contributions of the two stream communities to the sink communities were quantified.

A co-occurrence network was constructed using the “igraph” and “Hmisc” packages in R. The OTUs were those that occupied at least 20% of the samples that had been used to construct the co-occurrence network. The pairwise Spearman's correlations between OTUs were calculated. Each shown connection has a correlation coefficient > |0.8| and a *P* value < 0.01. The pairwise comparisons based on OTUs and the false discovery rate (FDR)-adjusted *P* value were performed using the “rcorr” function in the “Hmisc” package. The co-occurrence network was visualized using Gephi platform (v. 0.9.2) (https://gephi.org) (Bastian et al. 2009). In addition, we performed indicator species analysis of whole OTUs based on the OTU relative abundance (indval value > 0.6 and *P* < 0.05 are strong indicators for a habitat) using the indval function in R (version 4.0.3). And using the results about habitats of indicator operational taxonomic, we colored the network. Statistical differences in node-level attributes between the matrices were determined using non-parametric Kruskal–Wallis tests.

## Results

### Physiochemical characteristics

The physiochemical parameters of the water samples from the lake and streams are summarized in Table S1. As shown in Table S1, the surface water of the lake is characterized by low temperature and low pH values, but high conductivity. The water temperature (9.91 ± 2.39°C), pH (8.35 ± 0.04), DOC (1.06 ± 0.75 mg/L) and TN (0.19 ± 0.07 mg/L) showed the lowest values in the surface water of the lake. However, Cond and TDS showed the highest mean values in the lake surface water (mean 9.64 ± 14.68 ms/cm and 9585.77 ± 14802.76 mg/L, respectively). The lowest values of Cond (0.42 ± 0.09 ms/cm) and TDS (208.96 ± 44.39 mg/L) were found in glacial stream water.

### Bacterial taxonomic composition and diversity

**Figure 1.**
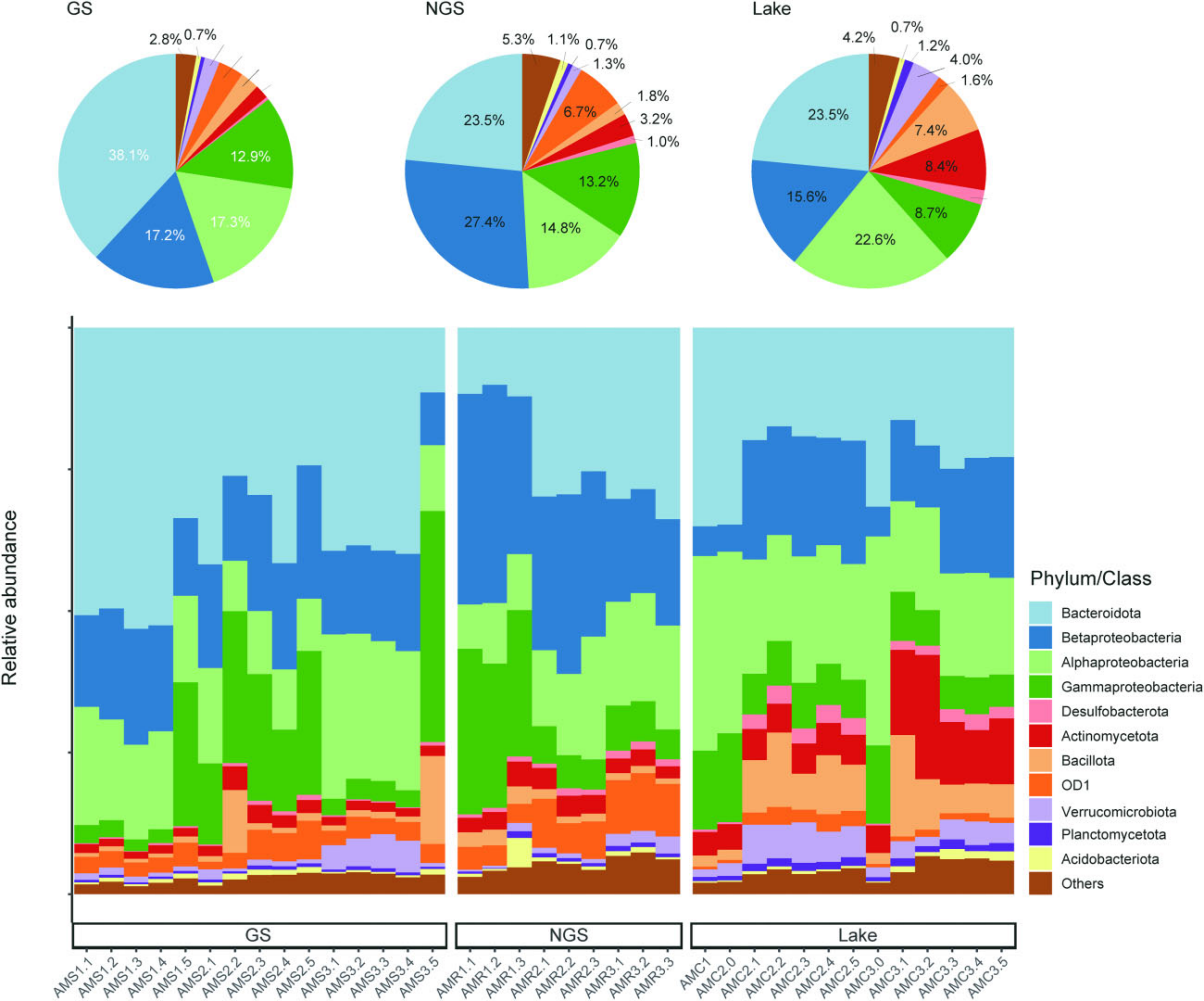
Relative abundance of dominant bacterial taxa at the phylum/class level. GS: the water samples of glacial stream; NGS: the water samples of non-glacial stream.

A total of 762 200 reads were obtained after quality control and rarefication, and these were clustered into 44 049 OTUs at 97% similarity level. Taxonomic analyses of our sequence data revealed that Pseudomonadota (original name: Proteobacteria) (mean relative abundance, 50%) and Bacteroidota (original name: Bacteroidetes) (28%) were the two dominant phyla in all stream and lake samples (Fig. 1). Ninety-five per cent of the Pseudomonadota phylum from the three habitats in total was related to the classes Betaproteobacteria, Alphaproteobacteria and Gammaproteobacteria, with Betaproteobacteria (27.4% of all sequences) and Gammaproteobacteria (13.2% of all sequences) enriched in the non-glacial stream. The Pseudomonadota populations were dominated by Alphaproteobacteria in the lake (22.6% of all sequences), and Desulfobacterota (original name: Deltaproteobacteria) were more abundant in the lake water samples than in stream samples. Actinomycetota (original name: Actinobacteria), Bacillota (original name: Firmicutes) and OD1 were present in low abundance in all samples (Fig. 1). Furthermore, we used LEfSe analysis to identify specialized bacterial communities of different habitats and search for biomarkers from the phylum to the genus level. A total of 37 microbial taxa were found to be significantly different among three habitats (Fig. S2). There were greater numbers of species enriched at a significant level (LDA > 4) in GS (15) and Lake (18) compared with those in NGS (4). Lefse analysis revealed that within the phyla Bacteroidota, the genera *Flavobacterium, Acinetobacter, Leadbetterella, Emticicia* and *Mycoplana* were enriched in the glacial stream samples, whereas the phylum OD1 and the genus *Pseudomonas* were enriched in the non-glacial stream samples. The dominant bacteria in the lake water samples were Actinomycetota, Bacillota and Verrucomicrobiota (original name: Verrucomicrobia). On the genus level, the *Planomicrobium* and *Luteolibacter* were enriched in the lake water samples.

**Figure 2.**
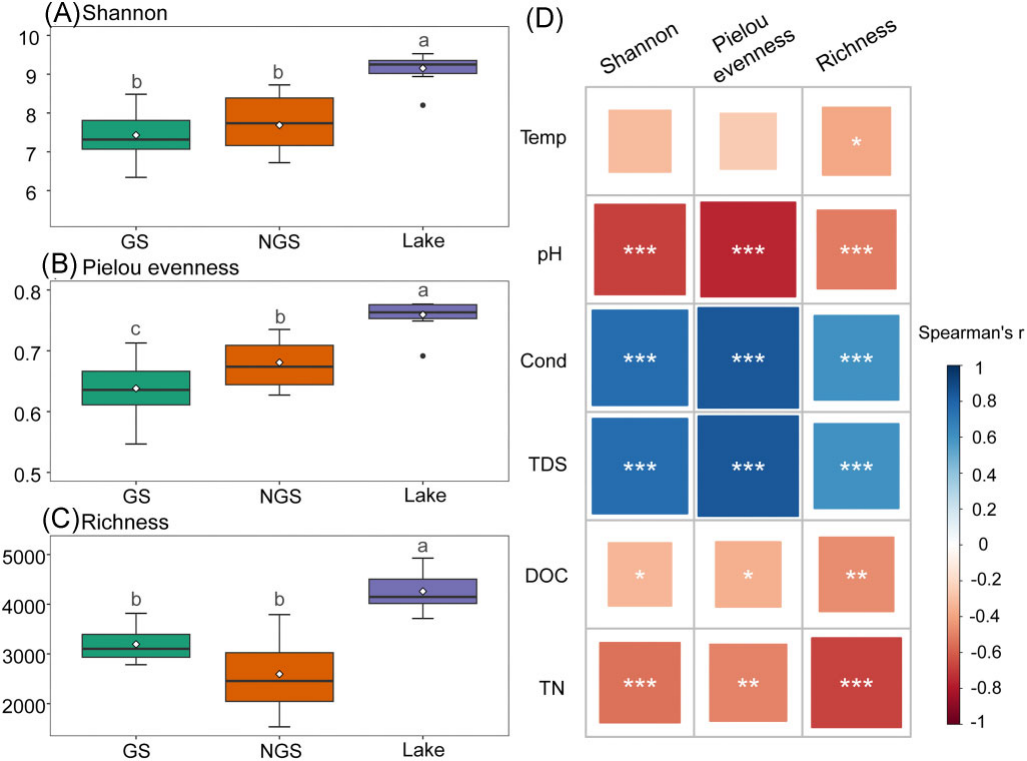
The bacterial alpha diversity among three habitats. Indices of alpha diversity are shown as Shannon diversity (A), Pielou's evenness (B) and Richness (C). Different lowercase letters indicate significant differences among habitats. The top and bottom boundaries of each box indicate the 75th and 25th quartile values, respectively, and lines within each box represent the median values. The mean values are represented by the white point. (D) Spearman correlations between the alpha diversity and environmental variables. Asterisks indicate the statistical significance (***P < 0.001; **P < 0.01; and *P < 0.05). GS refers to communities collected from water samples of the glacial stream. NGS refers to communities collected from water samples of the non-glacial stream. Temp: temperature; Cond: conductivity; TDS: total dissolved solids; DOC: dissolved organic carbon; TN: total nitrogen.

The Shannon diversity index ranged from 6.3 to 9.5 across all samples, with the highest values in the surface water of the lake and the lowest in the glacial stream. Pielou's evenness estimated for all habitats ranged from 0.5 to 0.8, with the highest values in the surface water of the lake and the lowest in the glacial stream. The Richness for three habitats varied from 1533 to 4928, with mean values being highest in the lake surface water and lowest in the non-glacial stream. The alpha diversity indices of lake water samples were significantly higher than those in the two external inlet streams (Kruskal–Wallis test, all *P* < 0.05, Fig. 2).

**Figure 3.**
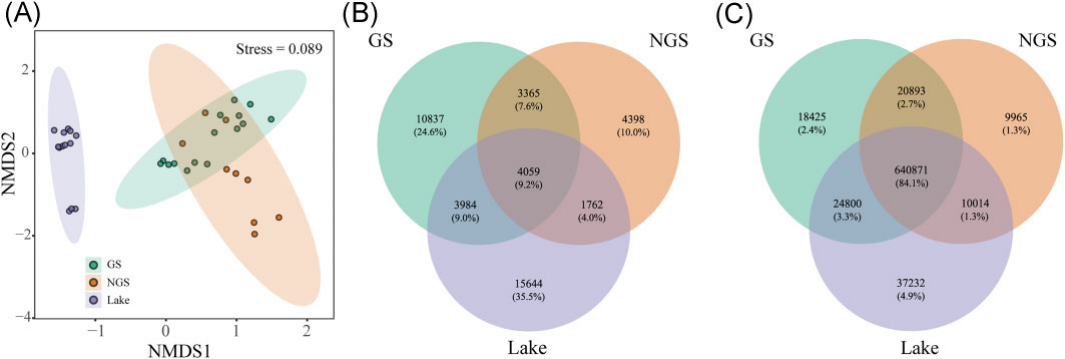
(A) Non-metric multidimensional scaling (NMDS) ordination visualization of bacterial community compositions (Bray–Curtis distance) among four habitats. The 95% confidence ellipses are shown for each habitat. Venn diagrams showing (B) the number and proportion of shared and unique OTUs and (C) the number and proportion of shared and unique sequences across the GS, NGS and Lake. GS: the water samples of the glacial stream; NGS: the water samples of the non-glacial stream.

NMDS analysis revealed the samples from the lake water and stream water were clearly separated (Fig. 3A). However, two stream samples partially overlapped to a certain extent. This observation was further confirmed by ANOSIM, showing that the microbial community structures were significantly different among habitats (Table S2). Lake water samples were found to be significantly dissimilar to glacial stream (R = 0.752, *P* < 0.001) and non-glacial stream samples (R = 0.742, *P* < 0.001). However, glacial stream samples were calculated to have low dissimilarities to non-glacial stream assemblages (R = 0.288, *P* < 0.01).

OTUs that were shared between sample groups were used as an indicator of the potential transfer of assemblages among habitats. The Venn diagrams indicated a total of 4059 OTUs (9.2% of the detected OTUs) with 84.1% (640 871 sequences) of total sequences that were shared among all three habitats (Fig. 3B and C). The proportion of unique OTUs was highest in the lake (35.5%, 15 644 OTUs), followed by the glacial stream (24.6%, 10 837 OTUs) and non-glacial stream (10.0%, 4398 OTUs). The number of OTUs shared between the glacial stream and the lake was 8043 (18.2%), which was higher than the OTUs between other pairwise habitats (Fig. 3B and C).

Sequences belonging to Bacteroidota (31.1%), Betaproteobacteria (21.3%) and Alphaproteobacteria (18.8%) dominated the shared OTUs of the three habitats (Fig. S3a). Sequences belonging to Bacteroidota (25.9%) and Alphaproteobacteria (22.8%) were the major OTUs shared between the glacial stream and lake water (Fig. S3b), while Alphaproteobacteria (23.0%) and Gammaproteobacteria (16.4%) were the major OTUs shared between the non-glacial stream and lake water (Fig. S3c). Sequences assigned to the phylum Bacteroidota comprised the majority of taxa unique in the glacial stream (26.1%) and the lake (23.3%), followed by Alphaproteobacteria (15.0% and 18.4%, respectively) (Fig. S3d and f). Alphaproteobacteria, Gammaproteobacteria and OD1 dominated the unique OTUs in the non-glacial stream, accounting for 14.7%, 14.0% and 13.3% of all unique sequences, respectively (Fig. S3e).

**Figure 4.**
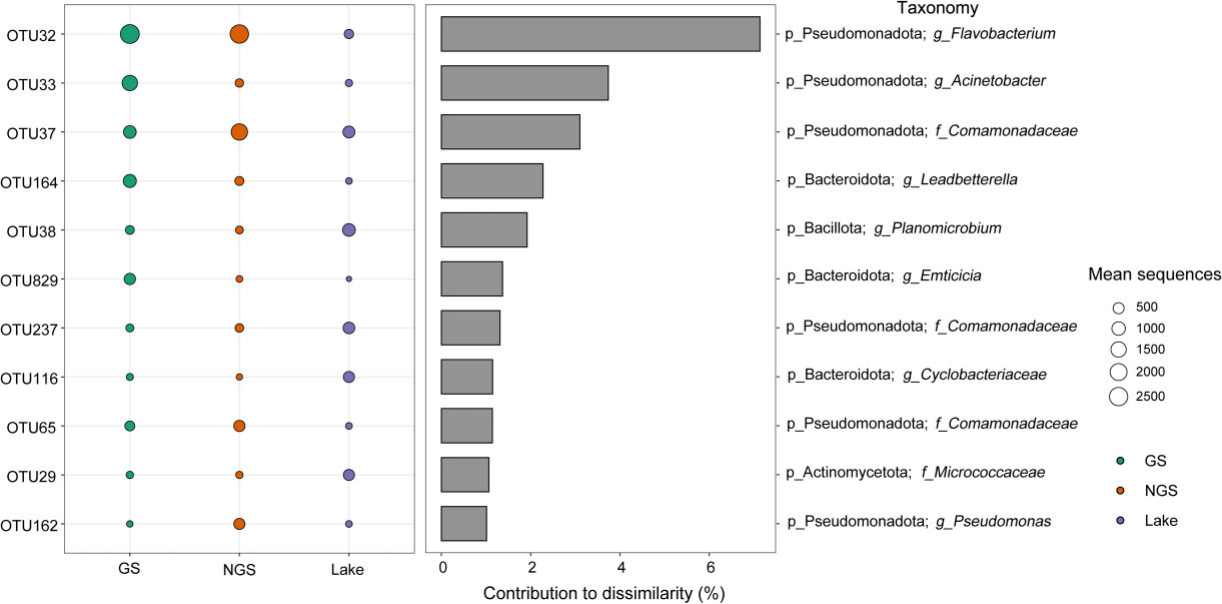
The SIMPER analysis showing the principal operational taxonomic units (OTUs) responsible for the differences between habitats. Mean sequences indicate the number of sequences belonging to each OTU. GS: the water samples of the glacial stream; NGS: the water samples of the non-glacial stream.

The SIMPER analysis identified the top 11 OTUs that cumulatively contributed 25.21% to the differences in bacterial community composition among three habitats (Fig. 4). The top 11 OTUs were dominated by species from the phyla Pseudomonadota and Bacteroidota, which together contributed 22.23% to the overall differences. The OTU32 belonging to the genus *Flavobacterium* was abundant in three habitats, explaining 7.14% of the overall community dissimilarity, followed by *Comamonadaceae* OTU37, OTU237 and OTU65 (5.56%) and *Acinetobacter* OTU33 (2.83%) (Fig. 4 and Table S3). The randomforest model revealed that pH, Cond and TDS were strong predictors for differences in relative abundances of most specific OTUs (Fig. S4). For example, pH was most correlated with the relative abundance of bacteria including *Flavobacterium* OTU32, *Cyclobacteriaceae* OTU116, *Comamonadaceae* OTU65 and *Micrococcaceae* OTU29. Cond and TDS were significantly related to *Flavobacterium* OTU32, *Planomicrobium* OTU38, *Emticicia* OTU829, *Cyclobacteriaceae* OTU116, *Comamonadaceae* OTU65 and *Micrococcaceae* OTU29.

### Linkage of environmental variables and bacterial biodiversity

Bacterial alpha diversity indices, including Shannon diversity, Richness and Pielou's evenness, exhibited strongly negative correlations with pH, DOC and TN, respectively (Spearman's rank correlations, *P* < 0.05 in all cases, Fig. 2D). However, Cond and TDS showed significantly positive relationships with three bacterial alpha diversity indices (Fig. 2D). For community composition, DistLM analysis showed that pH, Cond, TDS, Temp and TN were significantly correlated with bacterial community (Table 1). These significant variables totally explained 36.2% of the variation of the bacterial community composition. Of all the measured variables, pH (14.1%) was primarily responsible for the variation of the bacterial communities across the three habitats, followed by Cond (7.7%), TDS (5.3%), Temp (4.6%) and TN (4.6%).

**Table 1.** Distance-based multivariate linear model of bacterial community composition showing percentage of variation explained by environmental variables. (Bray–Curtis distances, 999 permutations.)

Variable	pseudo-F	*P*	Percentage variation explained	Cumulative variation explained
pH	5.727	**0.001**	0.141	0.141
Cond	3.341	**0.001**	0.077	0.218
TDS	2.395	**0.001**	0.053	0.271
Temp	2.159	**0.001**	0.046	0.317
TN	2.209	**0.001**	0.046	0.362
DOC	0.989	0.471	0.020	0.382

Data in bold represent significant correlations (*P* < 0.05)

TDS: total dissolved solids; Cond: conductivity; TN: total nitrogen; Temp: temperature; DOC: dissolved organic carbon.

### Source contributions

**Figure 5.**
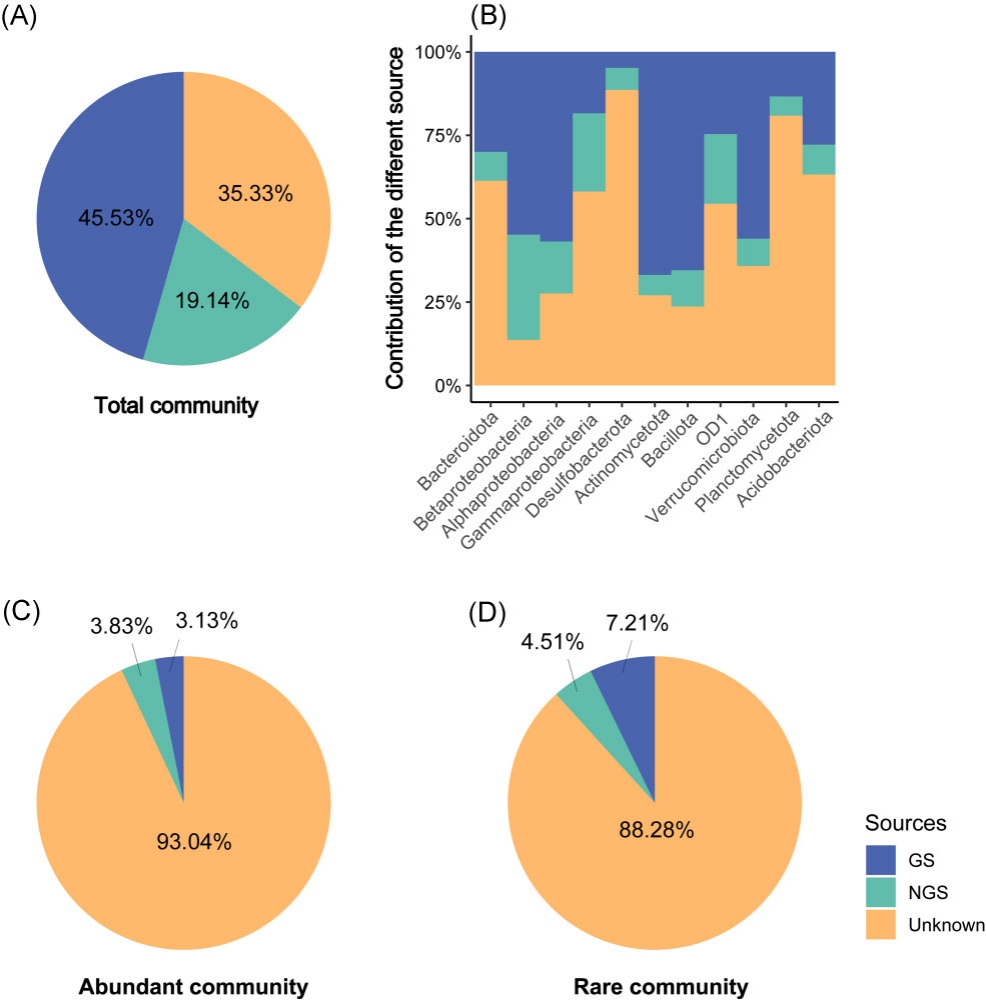
Results from SourceTracker analysis showing the contribution of the different source communities to the lake water. (A) Based on the total OTUs. (B) Based on the 11 most abundant phyla/classes. (C) Based on the abundant OTUs. (D) Based on the rare OTUs. GS: the water samples of the glacial stream; NGS: the water samples of the non-glacial stream.

Using the Bayesian classifier SourceTracker, we evaluated the importance of two inlet stream sources of diversity for downstream lake communities (Fig. 5). SourceTracker results revealed that 64.67% of the total community in the lake water was seeded by the glacial streams (45.53%) and the non-glacial streams (19.14%) microbial communities (Fig. 5A). At the population level, stream sources contributed 86.35% and 76.34% of the total sequences of Betaproteobacteria and Bacillota in lake surface water communities, respectively. However, the potential contributing sources for Desulfobacterota (88.6%) and Planctomycetota (original name: Planctomycetes) (80.9%) in lake water communities were largely unknown (Fig. 5B). Furthermore, only 6.96% of the abundant community (Fig. 5C) and 11.72% of the rare community (Fig. 5D) in the lake water were contributed by glacial stream and non-glacial stream bacterial communities, respectively. The potential contributing sources were largely unknown (93.04% and 88.28%, respectively). As for the abundant communities, lake water bacterial communities derived from the glacial stream (3.13%) were lower than those from the non-glacial stream (3.83%) (Fig. 5C). However, the rare communities showed the opposite pattern, with the glacial stream (7.21%) being a relatively significant source for lake water bacterial communities (Fig. 5D).

### Co-occurrence patterns of bacterial communities in different habitats

**Figure 6.**
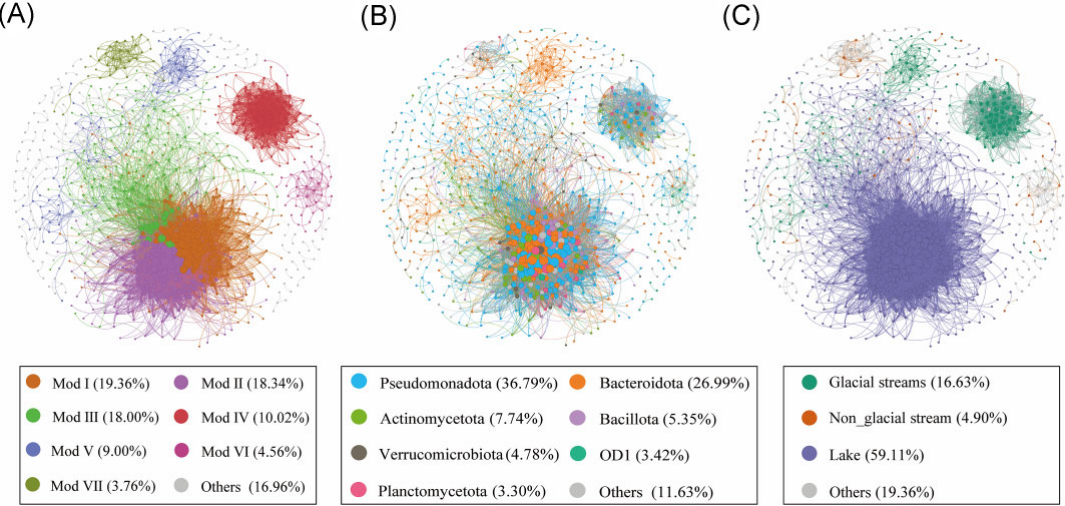
Co-occurrence networks of the bacterial community based on pairwise Spearman's correlations between OTUs. A connection denotes a strong (Spearman’s ρ > 0.8) and significant (*P* < 0.01) correlation. The nodes in the networks are colored according to modularity class (A), taxonomy (B) and habitats (C). Node size is proportional to the number of connections (i.e. degree).

Co-occurrence patterns of bacterial taxa were estimated by constructing correlation networks for water samples from different habitats. The correlation-based network consisted of 878 nodes (OTUs) and 16 880 edges (correlations) for the whole bacterial communities. A module is defined as a group of OTUs that are linked more tightly together. Here, bacterial networks were clearly parsed into seven major modules, of which modules I, II and III accounted for 19.36%, 18.34% and 18.00% of the whole bacterial network, respectively (Fig. 6A). The nodes in the network were assigned to 24 bacterial phyla, among which three phyla (Pseudomonadota, Bacteroidota, Actinomycetota) were widely distributed, accounting for more than 71.52% of all nodes (Fig. 6B).

The network was colored in accordance with the indicator species of three habitats; we found these nodes showed different preferences to habitats (Fig. 6C). For example, the majority of nodes in module 1, module 2 and module 3 were the most abundant in lake water samples, the majority of nodes in module 4 and module 5 were the most abundant in glacial streams samples, while nodes in module 7 were the most abundant in non-glacial streams samples. This finding suggests that habitat difference plays a key role in determining the network modular structure. Bacterial communities within each habitat could have more interactions instead of outside it.

To further evaluate the interactions of OTUs within a network of three habitats, their corresponding node-level topological properties were calculated (Fig. S5). In general, the higher the values, the stronger the interactions of species (Banerjee et al. 2018). Values of the topological features including node degree (connectivity), betweenness centrality and eigenvector centrality were highest in lake surface water samples, indicating that bacterial communities in the glacial-fed lake had the strongest co-occurrence associations (Kruskal–Wallis test: *P* < 0.05).

## Discussion

### Habitat-specific patterns of bacterial communities

Our finding indicated that the bacterial alpha diversity was significantly higher in the lake water than glacial and non-glacial streams (Fig. 2). This result was inconsistent with several studies that reported the bacterial alpha diversity was higher in inlet streams as opposed to surface water samples (Crump et al. 2012, Comte et al. 2017, Cavaco et al. 2019, Gu et al. 2021). A possible explanation for this might be that the inlet streams carry allochthonous microorganisms into the lake, thus we could consider the surface water of the lake as a “sink” of glacial and terrestrial ecosystems. These streams are likely passive conduits for microorganisms sourced from the upstream ecosystems (Cavaco et al. 2019, Zhang et al. 2021) and can be selectively seeded in the downstream lake ecosystems (Sheik et al. 2015, Hauptmann et al. 2016).

The bacterial communities showed clear separation between the three habitats (Fig. 3A). Pseudomonadota and Bacteroidota were dominant bacterial phyla in the lake and two streams of water (Fig. 1). The high relative abundances of Pseudomonadota and Bacteroidota observed in the glacial-fed lake water were not surprising as they have also been observed in many studies of glacier-fed lake ecosystems (Gu et al. 2021, Liu et al. 2021, Zhang et al. 2021). Interestingly, we found that the phylum OD1 presented a high relative abundance in the non-glacial stream water. The phylum Parcubacteria (OD1) belongs to the super phyla Patescibacteria, which has been found to be prevalent in water environments and streamlined many functions to adapt to the special environment, such as low and less nutrients, darkness and low oxygen (Tian et al. 2020).

Different habitat conditions probably lead to the percentage of shared OTUs among the three habitats being lower than those unique OTUs occurring in each habitat (Fig. 3B). However, the few shared OTUs (9.2%) accounted for a considerable proportion of the total sequences (84.1%), further substantiating the idea that the most ubiquitous taxa are often the most abundant (Fig. 3B and C) (Salazar et al. 2016, Li et al. 2023). Taxonomic composition analyses at the order level revealed that species affiliated with Burkholderiales (Betaproteobacteria) and Flavobacteriales (Bacteroidota) dominated the shared OTUs (Fig. S3a). These bacteria are well known for their organic matter degradation abilities and wide distribution in high-altitude lakes and/or stream ecosystems (Newton et al. 2011, Hotaling et al. 2019, Liu et al. 2021). The most abundant unique taxa identified in each habitat (e.g. Bacteroidota in glacial stream and lake) were similar to those dominant shared taxa across the three habitats. This finding is consistent with previous studies on some taxa displaying very different spatial distributions and environmental preferences and tolerances (Ruiz-Gonzalez et al. 2019).

SIMPER analysis identified 11 OTUs primarily responsible for the differences in bacterial community compositions observed across the three habitats (Fig. 4). These OTUs are strongly associated with environmental variables (Fig. S4). For instance, the discriminant taxa belonging to the genus *Flavobacterium* were negatively correlated with Cond and TDS, but positively correlated with pH and TN. Members of the *Flavobacterium* were highly abundant in freshwater and marine ecosystems, and were known for their ability to rapidly exploit bioavailable organic matter (Wakiewicz and Irzykowska 2014).

Our results also illustrated that distribution of bacterial communities was driven by local environmental variables. We found that pH was the critical environmental factor in shaping the distribution of bacterial communities across the three habitats (Table 1). The importance of pH in shaping microbial communities has been shown in previous studies conducted in lakes (Lindstrom et al. 2005, Ren et al. 2015). The pH could affect microbial growth and metabolism by altering the balance of H^+^ and OH^–^ ions on the cell wall/membrane (Yang et al. 2019). Our results further demonstrated that conductivity was another driving factor that differed between bacterial communities among the three habitats. Previous studies have shown that conductivity is a major parameter driving the community patterns of streams (Wilhelm et al. 2013) and lakes (Liu et al. 2019, Gu et al. 2021). The conductivity is a measure of water conduction current, which is related to the total dissolved salt content of the water. Aquatic microbes require a relatively stable concentration of the major dissolved ions in the water. Levels too high or too low may limit microbial survival (Liu et al. 2019).

### Bacterial communities exhibit distinctive co-occurrence patterns across three habitats

We further examined the associations between bacterial communities of the Lake Amuco and its two inlet streams. Network topological properties can reflect interactions between microbial species. For instance, the degree value describes the connectivity between OTUs in a network (Deng et al. 2012), and nodes with high betweenness centrality represent organisms that are important for maintaining the network (Zhu et al. 2019, Zhang et al. 2021). In addition, the closeness centrality value reflects how quickly information spreads from a given node to other reachable nodes, and eigenvector centrality is used to describe the degree of a central node that is connected to other central nodes (Deng et al. 2012). Our results indicated that lake water bacterial OTUs have the highest node degree, betweenness centrality and eigenvector centrality values (Fig. S5). This suggests that bacterial OTUs in the lake water exhibited closer interconnections than those in the glacial and non-glacial streams. A possible explanation for this is that stochastic ratios of bacterial communities were generally lower in Amuco lake than in stream habitats (Liu et al. 2021), indicating that the relative influence of deterministic processes on bacterial communities increased from inlet streams to lake water. Deterministic processes involve environmental filtering and biotic interactions (Liu et al. 2021, Yang et al. 2022). In our study, bacterial communities of lake water were more prominently affected by environmental filtering (e.g. lower water temperature), and had stronger biotic interactions, that is species co-occur more frequently in the lake water. Another possible explanation is that high microbial diversity in the lake water may result in strong microbial co-occurrence associations.

### Contribution of the two streams to bacterial communities in the lake water

Our results revealed that the glacial stream, rather than the non-glacial stream, was an important source of diversity for bacterial community in the glacial-fed lake water, supporting our first hypothesis that the glacial stream contributes more species to the microbial community of the downstream lake than the non-glacial stream. Several phyla were predominately calculated to be sourced from the glacial stream, such as Actinomycetota, Bacillota, Alphaproteobacteria, Verrucomicrobiota and Betaproteobacteria (Fig. 5B). These phyla are regularly identified in glacial environments (Wilhelm et al. 2013, Sharma et al. 2020, Zhang et al. 2021), suggesting that they may comprise cryophilic taxa, which is supported by cultivation-dependent approaches (Cheng and Foght 2007, Sherpa et al. 2018). A possible explanation for this result is the presence of resident communities in the lake. This finding was supported by previous observations, which reported that the most important source of bacteria in lakes was the resident community due to the importance of priority effects (Hanson et al. 2012, Comte et al. 2017). Consistent with this finding, higher relative abundances of Planctomycetota and Desulfobacterota were observed in the lake water than in the streams (Fig. 1). Another possible explanation is that other possible sources were not considered in our study, such as soil (Crump et al. 2012) and other strata of the lakes (Comte et al. 2017).

Our results also showed that the rare communities in lake water contributed by the glacial stream are higher than those from the non-glacial stream, while abundant communities show the opposite proportions. These findings suggest that glacial streams transport more rare taxa to lake water bacterial communities, while non-glacial streams may transport more abundant taxa to lake water. In addition, the lower Shannon index and higher Richness of the glacial stream compared with the non-glacial stream supports this deduction (Fig. 2). A previous study reported that some taxa exhibited a high relative abundance in glacial streams and their predominance may have resulted in their extremely low evenness and lowest Shannon index (Freimann et al. 2013, Liu et al. 2016). This may be interpreted as a result of a dominance of a few rare taxa adapted to the current set of environmental conditions (Freimann et al. 2013). Once the glacial stream has decreased and shifted towards the non-glacial stream, rare taxa transported to lake water will subsequently be diminished. In addition, the water flow rate changes of the glacial stream across the season will directly and indirectly change the bacterial community composition. Bacterial community composition of the lake can be related to water flow and the import of bacterial cells from the drainage area. The water flow rate, to some extent, likely also mirrors water residence time in the stream, although the latter was not directly measured in this study. The importance of water residence time in shaping microbial communities has been shown in previous studies (Lindstrom et al. 2005, Ruiz-Gonzalez et al. 2015), as it can regulate the balance between the transportation of bacteria from adjacent ecosystems, and the sorting of species by local environmental conditions. These findings confirm that the glacial stream is important and represents a diverse source community for the glacier-fed lake (Wilhelm et al. 2013, Peter and Sommaruga 2016).

## Conclusions

In summary, this study provides insights into the distribution patterns and drivers of bacterial communities in a glacier-fed lake in the central Tibetan Plateau. Our results demonstrated that the bacterial communities exhibited high heterogeneity of the glacier-fed lake and its two inlet streams that was closely related to environmental factors, such as pH, conductivity and total dissolved solids. Our results also indicated that the glacial stream was an important source of diversity for the lake surface bacterial communities and that bacterial alpha diversity was highest in the lake surface water. Furthermore, species co-occurrences were more frequent in the lake water. Collectively, the findings expand our knowledge of microbes in a glacier-fed aquatic ecosystem and offer valuable insights into the sources of diversity for glacier-fed lakes.

## Supplementary Material

fiae018_Supplemental_File
