# Supplementary material for: Habitat-specific patterns of bacterial communities in a glacier-fed lake on the Tibetan Plateau
Source: FEMS Microbiol Ecol. 2024 Feb 20;100(3):fiae018. doi: 10.1093/femsec/fiae018 (PMC10903976; doi:10.1093/femsec/fiae018)
Supplement: fiae018_Supplemental_File [file fiae018_supplemental_file.docx]

**Supporting materials:**

**Habitat-specific patterns of bacterial communities in a glacier-fed lake on the Tibetan Plateau**

Xuezi Guo^1,2^, Qi Yan^3^, Feng Wang^1^, Wenqiang Wang^3^, Zhihao Zhang^1,2^, Yongqin Liu^1,3^, and Keshao Liu^1^*

*^1^* *State Key Laboratory of Tibetan Plateau Earth System, Environment and Resources (TPESER), Institute of Tibetan Plateau Research, Chinese Academy of Sciences, Beijing 100101, China*

*^2^* *University of Chinese Academy of Sciences, Beijing, China*

*^3^* *Center for the Pan-Third Pole Environment, Lanzhou University, Lanzhou, China*

**Running title:** Bacterial communities in a glacier-fed lake

**Table S1.** Mean values (±SD) of the environmental characteristics of the three habitats.

|  | GS | NGS | Lake |
| --- | --- | --- | --- |
| Temp (℃) | 14.27±7.34 **ab** | 15.00±0.16 **a** | 9.91±2.39 **b** |
| pH | 9.89±0.38 **a** | 9.53±0.06 **b** | 8.35±0.04 **c** |
| Cond (ms/cm) | 0.42±0.09 **c** | 0.81±0.02 **b** | 9.64±14.68 **a** |
| TDS (mg/L) | 208.96±44.39 **c** | 402.89±9.32 **b** | 9585.77±14802.76 **a** |
| DOC (mg/L) | 4.80±6.25 **a** | 3.57±3.05 **a** | 1.06±0.75 **b** |
| TN (mg/L) | 0.38±0.21 **a** | 0.45±0.15 **a** | 0.19±0.07 **b** |

Means ± standard deviation.

Data in the same raw followed by same characters letters indicated no significant changes (P > 0.05).

GS: the water samples of glacial stream; NGS: the water samples of non-glacial stream.

Temp : temperature; Cond : conductivity; TDS : total dissolved solids; DOC : dissolved organic carbon; TN : total nitrogen.

**Table S2.** Table of ANOSIM analyses comparing sample group pairs.

| Habitats | ANOSIM | | |
| --- | --- | --- | --- |
|  | r | P | Permutations |
| GS-NGS | 0.288 | 0.007 | 9999 |
| GS-Lake | 0.752 | 0.001 | 9999 |
| NGS-Lake | 0.742 | 0.001 | 9999 |
| Global | 0.633 | <0.001 | 9999 |

GS: the water samples of glacial stream; NGS: the water samples of non-glacial stream.

**Table S3.** Table of SIMPER analyses showing the top contributing OTUs to Bray-Curtis distances of bacterial communities across three habitats.

| OTU ID | Relative  abundance | Occupancy | Phylum | Class | Order | Family | Genus |
| --- | --- | --- | --- | --- | --- | --- | --- |
| OTU32 | 8.60% | 1 | Bacteroidota | Flavobacteriia | Flavobacteriales | *Flavobacteriaceae* | *Flavobacterium* |
| OTU33 | 3.19% | 1 | Pseudomonadota | Gammaproteobacteria | Pseudomonadales | *Moraxellaceae* | *Acinetobacter* |
| OTU37 | 4.80% | 1 | Pseudomonadota | Betaproteobacteria | Burkholderiales | *Comamonadaceae* | */* |
| OTU164 | 2.06% | 1 | Bacteroidota | Cytophagia | Cytophagales | *Cytophagaceae* | *Leadbetterella* |
| OTU38 | 1.83% | 1 | Bacillota | Bacilli | Bacillales | *Planococcaceae* | *Planomicrobium* |
| OTU829 | 1.11% | 0.84 | Bacteroidota | Cytophagia | Cytophagales | *Cytophagaceae* | *Emticicia* |
| OTU237 | 1.42% | 1 | Pseudomonadota | Betaproteobacteria | Burkholderiales | *Comamonadaceae* | */* |
| OTU116 | 0.98% | 1 | Bacteroidota | Cytophagia | Cytophagales | *Cyclobacteriaceae* | */* |
| OTU65 | 1.29% | 1 | Pseudomonadota | Betaproteobacteria | Burkholderiales | *Comamonadaceae* | */* |
| OTU29 | 1.04% | 1 | Actinomycetota | Actinobacteria | Actinomycetales | *Micrococcaceae* | */* |
| OTU162 | 0.70% | 1 | Pseudomonadota | Gammaproteobacteria | Pseudomonadales | *Pseudomonadaceae* | *Pseudomonas* |


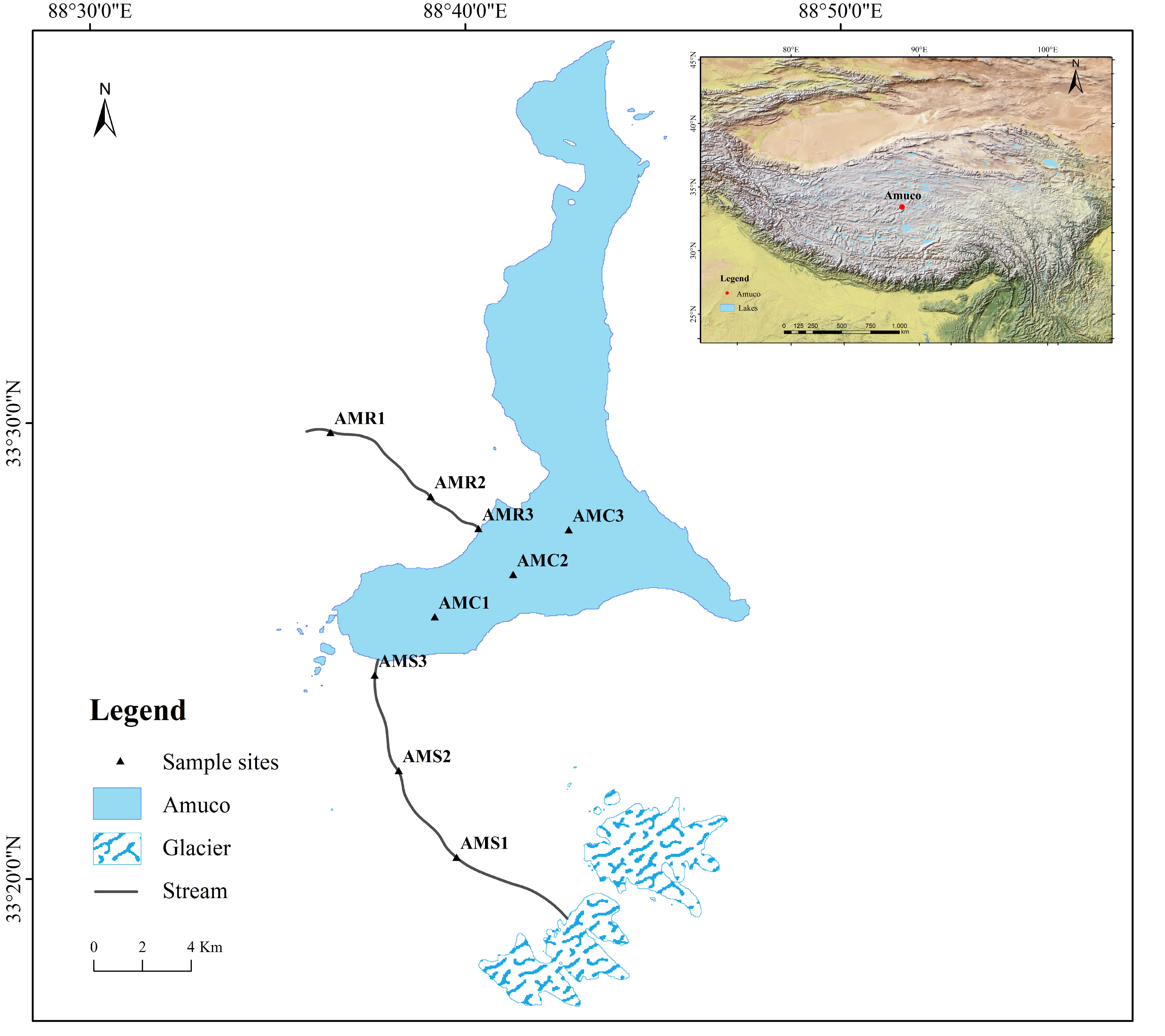


**Fig. S1.** Geographical location of the sampling sites in Lake Amuco (AMC1-AMC3), glacial stream (AMS1-AMS3) and non-glacial stream (AMR1-AMR3).

**
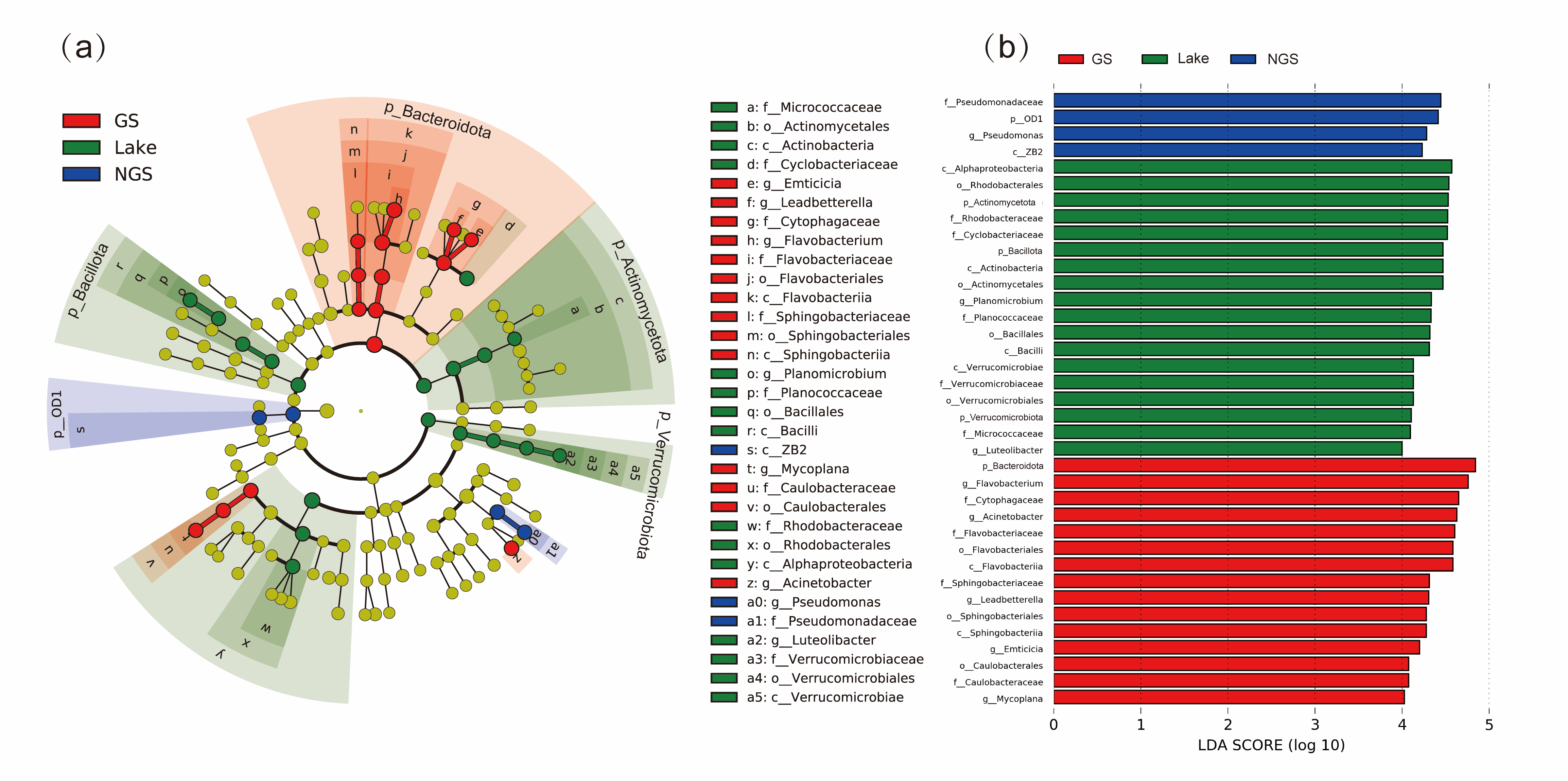
**

**Fig. S2.** LEfSe analysis of 16S rRNA gene sequences displaying the differences in water microbial communities among GS, NGS and Lake habitats. (a) The cladogram indicates the distribution of water microbial lineages with different types of habitats. Each circle’s size is proportional to the taxon’s abundance. (b) Histogram of linear discriminant analysis (LDA) scores for differentially abundant microbiota (LDA > 4). Differences are represented by colors indicating the most abundant class (red = water samples of glacial stream, green = water samples of lake, blue = water samples of non-glacial stream, yellow = not significant).

**
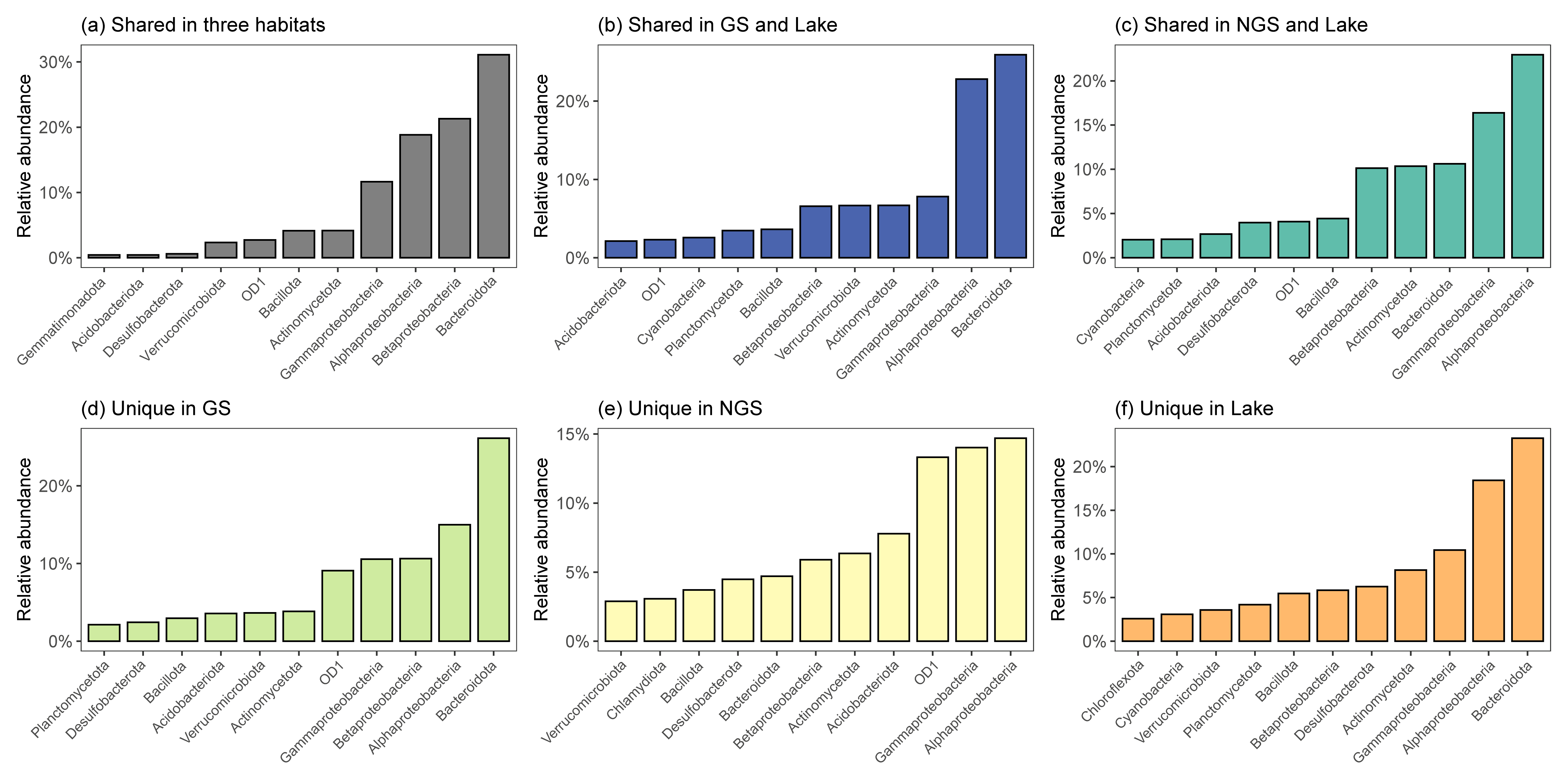
**

**Fig. S3.** Distribution of abundant taxonomic groups in lake and two streams. (a) Top 11 abundant bacterial phyla/classes shared across the GS, NGS and Lake. (b-c) Top 11 abundant bacterial phyla/classes shared between GS/NGS and Lake. (d-f) Top 11 abundant bacterial phyla/classes unique in the GS, NGS and Lake, respectively. GS: the water samples of glacial stream; NGS: the water samples of non-glacial stream.


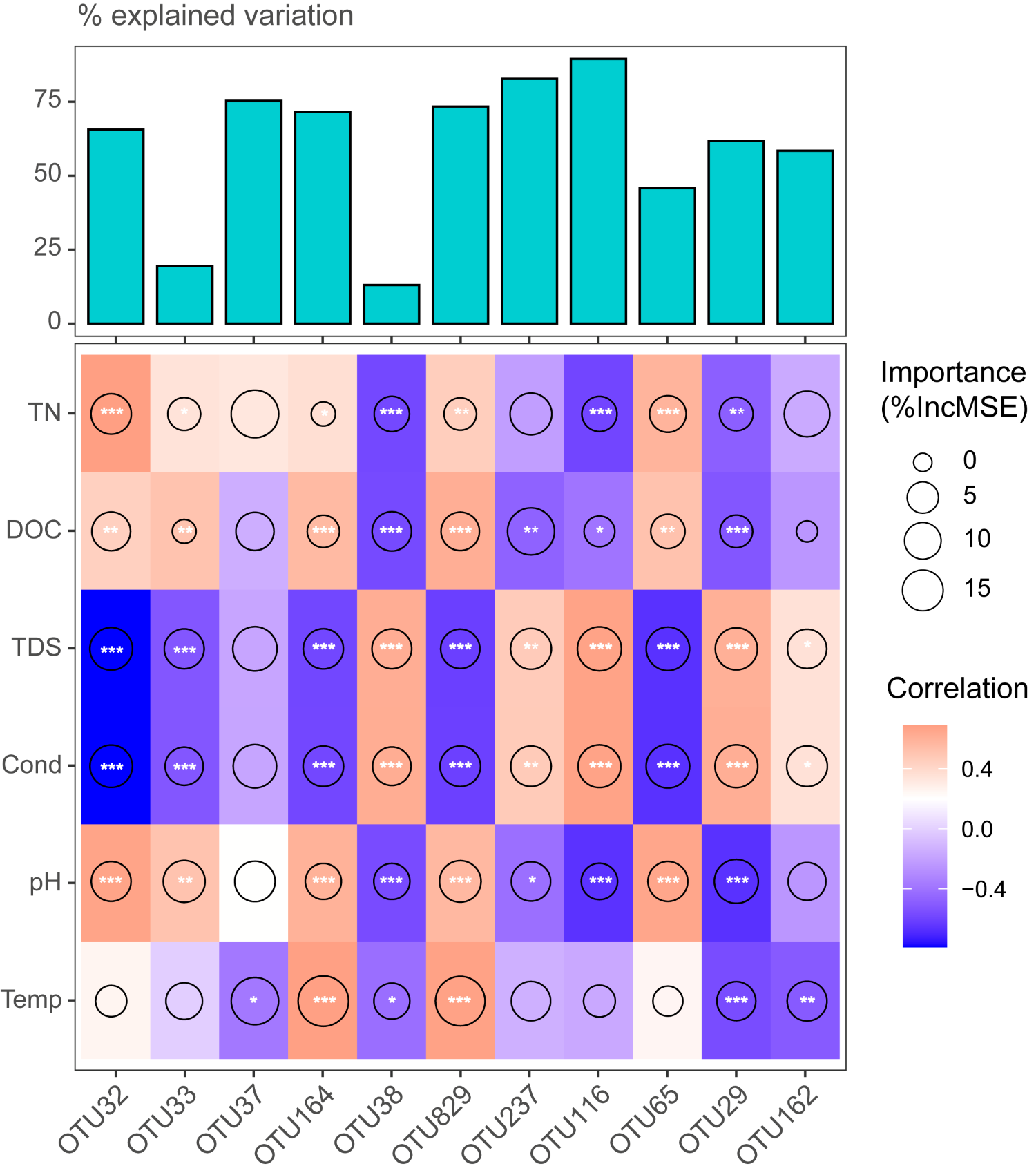


**Fig. S4.** Contributions of environmental factors to the dissimilarities in relative abundances of top 11 OTUs based on correlation and best multiple regression model. Circle size represents the variable importance. Colors represent Spearman correlations. Asterisks indicate the statistical significance (****P* < 0.001; ***P* < 0.01; and **P* < 0.05). The abbreviations of environmental factors accorded to the Environmental parameter.


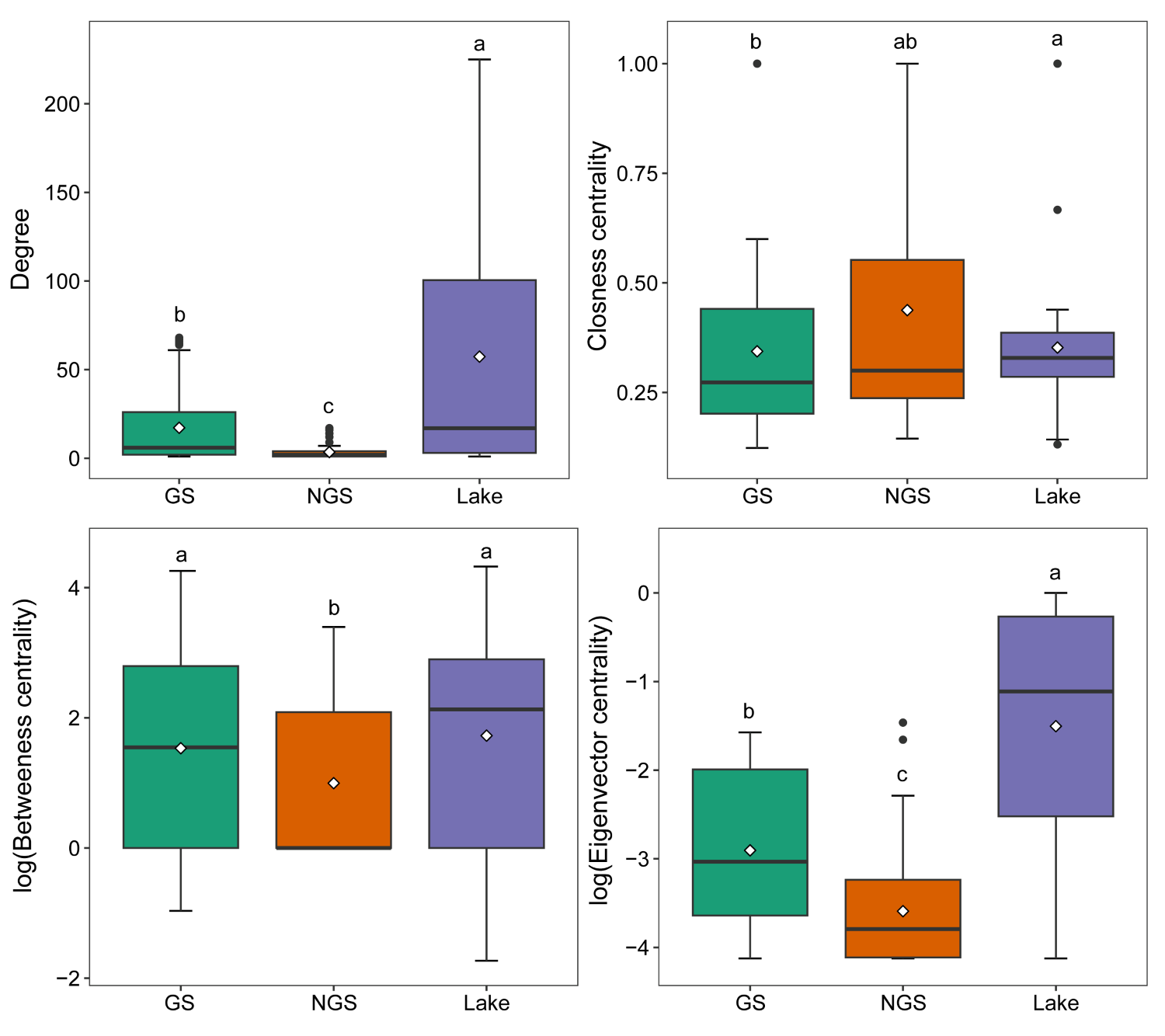


**Fig. S5.** Multiple network node properties of bacterial co-occurrence networks, including node degree, closeness centrality, betweenness centrality, and eigenvector centrality. Different lowercase letters indicate significant differences among habitats.

GS: the water samples of glacial stream; NGS: the water samples of non-glacial stream.
